# Motivational deficits and anhedonia in schizophrenia: insights from behavioral activation and behavioral inhibition systems

**DOI:** 10.1186/s12991-026-00667-0

**Published:** 2026-05-10

**Authors:** Özge Akgül, Emine Ilgın Hosgelen, Taha Selim Bilgin, Berna Binnur Akdede, Köksal Alptekin

**Affiliations:** 1 Faculty of Arts and Sciences, Department of Psychology, İzmir Democracy University, İzmir, Turkey; 2 Department of Neurosciences, Dokuz Eylul University, İzmir, Turkey; 3 Department of Psychiatry, Health Sciences University- İzmir Tepecik Education and Research Hospital, İzmir, Turkey; 4 Department of Psychiatry, Dokuz Eylul University, İzmir, Turkey

**Keywords:** Behavioral activation, Behavioral inhibition, Anhedonia, Schizophrenia, Depressive symptoms

## Abstract

**Purpose:**

Schizophrenia is a disorder characterized by significant motivational deficits, including anhedonia, that impair daily functioning. Approach and avoidance behaviors are regulated by two basic motivation systems: the Behavioral Inhibition System (BIS) and the Behavioral Activation System (BAS). Based on Gray’s Reinforcement Sensitivity Theory, this study examined the relationships among BIS/BAS activity, depressive symptoms, and anhedonia in individuals with schizophrenia compared to healthy controls.

**Methods:**

Sixty-one patients with schizophrenia (SZ) and 62 healthy participants participated in the study. Positive and Negative Syndrome Scale, Beck Depression Inventory, Calgary Depression Scale, BIS/BAS Scale, and physical, social, and chemosensory anhedonia scales were administered to the participants. This cross-sectional, between-groups study compared SZ and healthy controls to examine the associations among BIS/BAS activity, depressive symptoms, and anhedonia. T-test, Logistic regression, and regression analyses were performed.

**Results:**

Logistic regression analyses determined that BAS activity and anhedonia significantly predicted being in the patient group. It revealed motivational deficits in schizophrenia compared to healthy subjects. Patients with schizophrenia showed reduced BAS activity associated with impaired reward sensitivity and goal-directed behavior. However, no differences in BIS activity were found. While BAS activity and depressive symptoms were found to be predictors of all subscales of anhedonia in healthy controls, they were only found to be predictive of physical anhedonia in schizophrenia.

**Conclusion:**

These results suggest that anhedonia in SZ results from deficits in anticipatory pleasure and reward processing, independent of mood-related mechanisms. It suggests that different mechanisms may underlie anhedonia. Future studies should investigate BAS dysregulation in SZ, including cognitive-behavioral therapies and pharmacological strategies that target dopaminergic pathways.

## Introduction

Motivational and reward-processing abnormalities are central features of schizophrenia and contribute significantly to its affective and functional outcomes. According to Gray’s Reinforcement Sensitivity Theory (RST), individual differences in motivation and affective behavior can be understood through two primary neurobehavioral systems: the Behavioral Activation System (BAS), which governs approach and reward-seeking behavior, and the Behavioral Inhibition System (BIS), which regulates avoidance and sensitivity to punishment [1]. Alterations in these systems have been implicated in both schizophrenia and mood disorders, suggesting that disruptions in approach–avoidance balance may underlie anhedonia and motivational deficits observed in schizophrenia [2].

Anhedonia, which is defined as a diminished capacity to experience pleasure, is accepted as one of the core negative symptoms of schizophrenia (SZ). This symptom has gained substantial attention in recent years due to its strong association with motivational processes and functional impairments [3]. The growing body of studies has increasingly distinguished between two components of pleasure: consummatory pleasure, which refers to the immediate experience of pleasure, and anticipatory pleasure, which signifies the expectation or anticipation of future rewards [4]. Studies indicate that patients with schizophrenia do not have an impairment in their capacity to experience immediate pleasure, but they experience motivational impairments due to their deficit in goal-directed representations of future rewards [5].

Research Domain Criteria (RDoC) described anhedonia as a more transdiagnostic construct that can be seen in psychiatric disorders, including schizophrenia and affective disorders, linking with amotivation and apathy [6]. However, findings report that both the description and underlying mechanisms of anhedonia differ between these two disorders. For example, in depression, anhedonia points out a generalized reduction in both anticipatory and consummatory pleasure and is often accompanied by emotional blunting and reduced responsiveness to rewarding stimuli [7]. On the other hand, consummatory pleasure is mostly intact in SZ, although they report impairments in anticipatory pleasure [4, 8]. These differences may point out great clinical importance in understanding the subtypes of anhedonia. While anhedonia in SZ reflects impaired goal-directed behavior despite intact emotional capacity, depressive anhedonia is associated with emotional dysregulation [9]. These findings propose that anhedonia in schizophrenia is not a generalized affective flattening but a selective deficit in motivation-related pleasure. Rather than reflecting a general inability to feel pleasure, motivational deficits may underlie reduced goal-directed behavior in schizophrenia.

In recent years, RST has attracted researchers to understand motivational processes. According to Gray’s model (1994), prominent motivational models delineate two fundamental dimensions of motivation: behavioral approach and behavioral avoidance [1]. The BIS is activated by stimuli associated with punishment or non-reward, prompting individuals to avoid negative outcomes. Elevated BIS activation predisposes individuals to heightened sensitivity to loss and diminished reward responsiveness [10]. Conversely, BAS mediates responses to conditioned and unconditioned appetitive stimuli, facilitating behaviors aimed at reward acquisition or punishment avoidance. Individuals with heightened BAS activation demonstrate increased reward-seeking tendencies and engage more frequently in novel activities. Conversely, those with elevated BIS activation exhibit behavioral patterns consistent with heightened sensitivity to negative outcomes. To understand psychopathology, including schizophrenia, anxiety disorders, and depression, the BIS and BAS can be used as a transdiagnostic framework [2]. Because those systems are considered critical for comprehending how individuals regulate their behavior concerning environmental stimuli.

Regarding BAS, while some studies did not report any difference between HC and SZ [11], Tso et al. reported lower BAS in SZ [12]. Gard et al. found BAS but not BIS to be positively associated with anticipatory but not consummatory pleasure in schizophrenia [4, 13]. Especially for SZ, reduced anticipatory pleasure can undermine behavioral activation, as individuals may not be motivated to pursue goals if they cannot foresee the potential enjoyment or value of achieving them. Previous studies have found that individuals with schizophrenia show higher BIS scores compared to control participants, indicating elevated threat sensitivity [5, 11]. Elevated BIS is linked to greater social and emotional difficulties [11]. The BIS hyperactivity can lead to avoidance behaviors, increased social withdrawal, and higher levels of stress, exacerbating both positive and negative symptomatology. These findings suggest that reward processing deficits (low BAS) and heightened threat sensitivity (high BIS) may underlie key symptoms of schizophrenia, especially anhedonia and motivational impairments. The interaction between a hypoactive BAS and a hyperactive BIS may thus create a feedback loop, where diminished reward sensitivity and heightened punishment sensitivity jointly contribute to the persistence of negative symptoms [14]. However, despite these findings, the importance of BIS/BAS systems in understanding anhedonia has not been sufficiently explored in SZ. While these systems offer a promising transdiagnostic approach to understanding motivational disorders, studies directly examining their role in schizophrenia in relation to anticipatory and consummatory anhedonia are limited. Unlike previous studies conducted by Tso et al. [12] and Gard et al. [4, 13] which primarily examined the association between BIS/BAS functioning and anticipatory or consummatory pleasure, the present study integrates multiple dimensions of anhedonia (physical, social, and chemosensory) to provide a more comprehensive understanding of motivational deficits in schizophrenia. Moreover, this is the first study to apply the BIS/BAS framework to a Turkish clinical population, thereby extending existing findings to a different cultural and linguistic context. In addition, examining the predictive factors between depressive symptoms, BIS/BAS activity, and specific anhedonia domains allows for a more detailed assessment of the mechanisms underlying reward-related impairments in schizophrenia.

Specifically, this study examines the extent to which approach–avoidance motivational tendencies predict anhedonia symptoms in schizophrenia compared to healthy controls (HC). The present study aimed to examine the associations between BIS/BAS activity, depressive symptoms, and anhedonia in schizophrenia. We hypothesized that (1) patients with schizophrenia would differ from healthy controls in terms of BIS/BAS activity; (2) individuals with schizophrenia would exhibit significantly higher levels of anhedonia compared to healthy controls; (3) in individuals with schizophrenia, depressive symptoms and BIS/BAS will significantly predict different types of anhedonia (social, physical and chemosensory anhedonia); and (4) BIS/BAS activity and depressive symptoms would significantly predict group membership (schizophrenia vs. control). These hypotheses were developed to clarify the motivational mechanisms underlying hedonic deficits in schizophrenia.

By integrating a neuropsychological model of motivation with the phenomenological distinctions in anhedonia, this research seeks to contribute to a more nuanced understanding of negative symptoms in schizophrenia.

## Method

### Participants

The present study included a total of 61 patients diagnosed with schizophrenia (41 males, 20 females) according to DSM-V criteria, along with 62 healthy controls (32 males, 30 females). Participants were recruited from the Outpatient Schizophrenia Research Unit at Dokuz Eylul University Hospital. Inclusion criteria for the schizophrenia group required participants to: (1) be aged between 18 and 55 years; (2) have no history of drug abuse or brain injury; (3) be free of neurological disorders; (4) have completed a minimum of five years of education; (5) not have received electroconvulsive therapy within the preceding six months; and (6) have no comorbid conditions or intellectual disabilities. All patients were undergoing treatment with antipsychotic medications. For the control group, inclusion criteria stipulated the absence of any neurological or psychiatric disorders, no family history of psychiatric illnesses among first-degree relatives, no history of drug abuse or brain injury, and normal vision. Healthy control participants were recruited through public announcements at Dokuz Eylul University and Izmir Democracy University and social media postings.

This study was conducted in accordance with the ethical standards of the institutional research committee and with the 1964 Declaration of Helsinki and its later amendments or comparable ethical standards. Ethical approval was obtained from the Dokuz Eylul University Scientific Research and Publication Ethics Committee (Approval Date: 11.05.2022; Approval Number: GOAEK 7207). Informed consent was obtained from all participants prior to data collection, and all data were collected anonymously to ensure participant confidentiality.

### Procedure

Various scales and assessment tools were employed to evaluate the study hypothesis. The Sociodemographic Form, developed by the researchers, was used to collect information regarding participants’ demographic characteristics (e.g., age, gender, marital status, educational level).

#### Clinical measures

Positive and Negative Syndrome Scale (PANSS): The severity of positive and negative symptoms was assessed using PANSS on the day of evaluation [15]. The Turkish reliability and validity study was conducted by Kostakoglu et al. [16]. Patients who scored lower than 75 were considered to be stabilized [17].

The Calgary Depression Scale for Schizophrenia (CDSS): The Calgary Schizophrenia Depression Scale was administered to participants to assess depression in patients diagnosed with schizophrenia and to measure the level and severity of depressive symptoms [18]. The Turkish version of the scale was found to be reliable and valid [19]. CDSS was administered only to patients, as it was developed to assess depressive symptoms specific to schizophrenia while differentiating them from negative or extrapyramidal features [18].

Beck Depression Inventory (BDI): Healthy participants carried out the Beck Depression Inventory to evaluate symptoms of depression and their severity [20]. The Turkish reliability and validity of the scale were published by Hisli et al. [21]. It was used to capture subclinical depressive symptoms in a non-clinical population [22].

#### Behavioral inhibition and activation measurement tools

##### BIS/BAS scale

The BIS/BAS Scale, developed by Carver & White, consists of 4 subscales, namely “fun-seeking”, “reward sensitivity”, and “drive”, under the title of “behavioral inhibition system (BIS) subscale and behavioral activation system (BAS)”, and 24 items in total [10]. Since four of the 24 items in the scale are filler items, the evaluation is based on the remaining 20 items. Participants rate themselves on each item using a 4-point Likert-type scale (1 = Strongly agree, 2 = Somewhat agree, 3 = Somewhat disagree, 4 = Strongly disagree). Its validity and reliability in Turkish were assessed by Şişman [23]. The higher the score, the stronger the effect of the behavioral inhibition/activation system.

#### 2.2.3. Anhedonia measurement tools

To assess different domains of anhedonia, the following self-report scales were administered:

##### Physical anhedonia scale (PAS)

The Physical Anhedonia Scale was developed by Chapman and colleagues in 1976 [24]. Physical anhedonia encompasses sensory experiences including taste, tactile sensations, sexual experiences, temperature perception, movement, olfaction, and auditory stimuli. PAS is a 61-item self-report questionnaire, with each item requiring a “yes” or “no” response. Responses are scored on a binary scale (0 or 1), yielding a total score that can range from 0 to 61. Baskak et al. (2009) indicated that 50 out of 61 items were valid and reliable throughout the Turkish population [25].

##### Social anhedonia scale (SAS)

Chapman & Raulin initially developed the scale as a 48-item self-report instrument to assess social fulfillment and social anxiety [24]. Mislove & Chapman documented the psychometric characteristics of the definitive 40-item version. The validity and reliability study in Turkish was conducted by Cihan et al. in 2015 [26].

##### Chemosensory pleasure scale (CPS)

The Chemosensory Pleasure Scale is a self-reported instrument that evaluates an individual’s enjoyment derived from olfactory and gustatory stimuli [27]. It has three elements (food, imagination, and pure nature). The investigation on Turkish validity and reliability was conducted by Akgül et al. [28]. A low score on the Chemosensory Pleasure Scale (CPS) implies severe chemosensory anhedonia.

### Statistical analysis

A priori power analysis was conducted using G*Power version 3.1.9 to determine the minimum sample size required to detect group differences based on the effect size reported in Li et al. [29], Parameters were set at α = 0.05, power (1 − β) = 0.95, and Cohen’s *f* = 0.51 [29], yielding a required total of 110 participants (55 SZ, 55 HC). All data were screened for normality. Independent-samples *t*-tests were performed for continuous variables, and chi-square tests were used for categorical variables to examine demographic and clinical characteristics. All statistical analyses were conducted using IBM SPSS Statistics version 26.0.

*Preliminary analyses*

Independent-samples *t*-tests were initially performed to compare the SZ and HC groups on continuous variables and chi-square test was conducted on categorical variables. Pearson correlation analyses were then conducted to examine associations between anhedonia components and clinical characteristics (i.e., PANSS positive and negative scores, CDSS total for patients with schizophrenia and BDI for healthy controls) as well as behavioral activation and inhibition measures (BIS/BAS) for each group. Statistical significance was defined as *p* < 0.05 for all analyses.

*Regression analyses*

Linear regression analyses were used to identify predictors of anhedonia, and logistic regression analyses were performed to examine predictors of group membership (SZ vs. HC). Based on the study hypotheses, anhedonia and BAS were treated as independent predictors. Depressive symptoms (CDSS for SZ; BDI for HC) and BAS were entered into regression models to assess whether motivational (BIS/BAS) and affective (depressive) factors uniquely predicted anhedonia beyond depressive symptoms. Although BIS was included in the study design, it was excluded from the regression models because preliminary correlations revealed no significant associations between BIS scores and anhedonia measures (PAS, SAS, CPS).

*Hypothesis testing*

To test Hypotheses 1 and 2 (group differences in motivation and pleasure), *t*-tests were followed by correlation analyses assessing relationships among BIS/BAS, depressive symptoms, and anhedonia within each group. To test Hypotheses 3 and 4, separate linear regression analyses were performed for SZ and HC groups, with BAS and BDI as predictors of anhedonia (PAS, SAS, CPS). Finally, logistic regression analyses were conducted to determine which variables best predicted group membership (SZ vs. HC), entering BIS/BAS and anhedonia variables as successive predictor sets.

## Results

### Group characteristics

Demographic, clinical, and psychometric information for both groups is presented in Table 1. The control group was not formally matched to the schizophrenia group; however, the two groups were comparable in terms of age and gender distribution (*p* > 0.05). Differences were observed in education level and marital status (*p* < 0.001). The patient group had more anhedonia compared to the HC. Also, they had lower BAS scores (*p* < 0.05). (Table 1).

**Table 1:** Groups’ demographic, clinical, and psychometric profiles

	SZ (*n* = 61)	HC (*n* = 62)	*p*-value
Age	38.1 ± 9.7	35.4 ± 13.5	0.19
Gender	20 F/41 M	30 F/32 M	0.78
Education	12.1 ± 4.3	15 ± 4	< 0.001**
Marital status			
Married	8	25	< 0.001**
Unmarried	53	37	
Physical Anhedonia Scale	17.5 ± 8.2	13.3 ± 9.4	< 0.001**
Social Anhedonia Scale	15.9 ± 7.8	13.1 ± 8.2	0.05*
Chemosensory Anhedonia Scale	58.1 ± 10.5	57.1 ± 10.9	0.004*
Food	19.9 ± 4.5	20.7 ± 3.7	0.28
Imagination	17.9 ± 7.3	20.8 ± 6	0.02*
Nature	14.9 ± 3.4	15.5 ± 2.8	0.32
BIS	21 ± 3.6	22.03 ± 3.3	0.11
BAS	38 ± 7.5	42 ± 5.4	0.001**
Reward responsiveness	16.5 ± 3.3	18.4 ± 2	< 0.001**
Fun-seeking	11.1 ± 3	12.3 ± 2.3	0.017*
Drive	10.4 ± 3.3	11.3 ± 2.5	0.1
PANSS total score	56.15 ± 12.84		
PANSS subscale score			
Positive	14.02 ± 4.49		
Negative	14.49 ± 4.80		
General psychopathology	27.59 ± 5.94		
CDSS	3.34 ± 3.27		
BDI		10.8 ± 8	

Data are presented as mean ± standard deviation. PANSS= Positive and Negative Syndrome Scale; BIS= Behavioral Inhibition System; BAS= Behavioral Activation System CDSS= Calgary Depression Scale for Schizophrenia; BDI= Beck Depression Inventory

*p<0.05; **p<0.001

### 3.2. Correlation analysis

Findings of correlation analyses indicate that BIS/BAS and anhedonia are correlated with physical anhedonia in HC (*p* < 0.05) (Table 2). Moreover, BAS was associated with clinical symptoms like PANSS total, PANSS general, and CDSS in the SZ group (*p* < 0.05) (Table 3).

**Table 2:** The Correlation Matrix for Healthy Controls

		1.BIS	2.BAS	3.PAS	4.SAS	5.CPS	6.BDI
1	*r*	1	-0.41	0.30	-0.98	0.14	0.13
	p		0.75	0.02*	0.45	0.28	0.31
2	r		1	-0.36	-0.48	0.57	-0.31
	p			0.004*	< 0.001**	< 0.001**	0.015*
3	r			1	0.66	-0.29	0.44
	p				< 0.001**	0.02*	< 0.001**
4	r				1	-0.33	0.44
	p					0.009*	< 0.001**
5	r						-0.42
	p						< 0.001

BIS= Behavioral Inhibition System; BAS= Behavioral Activation System; PAS= Physical Anhedonia Scale; SAS= Social Anhedonia Scale; CPS= Chemosensory Pleasure Scale; BDI= Beck Depression Inventory

** *p* < 0.01

* *p* < 0.05

**Table 3:** The Correlation Matrix for Patients with Schizophrenia

		1.BIS	2.BAS	3.PAS	4.SAS	5.CPS	6.PANSS Tot	7.PANSS PS	8.PANSS NS	9.PANSS GS	10.CDSS
1.	*r*	1	0.41	0.07	0.06	0.15	0.1	0.15	0.03	0.08	0.1
	p		< 0.001**	0.62	0.63	0.24	0.47	0.25	0.8	0.56	0.46
2.	r		1	-0.18	0.13	0.25	0.30	0.24	0.14	0.32	0.35
	p			0.16	0.33	0.06	0.03*	0.06	0.3	0.013*	0.008*
3.	r			1	0.61	-0.32	0.23	0.10	0.35	0.15	0.24
	p				< 0.001**	0.011*	0.08	0.46	0.007*	0.21	0.11
4.	r				1	-0.17	0.26	0.14	0.33	0.14	0.16
	p					0.18	0.05	0.31	0.01*	0.28	0.23
5.	r					1	-0.06	-0.05	0.04	-0.11	0.11
	p						0.67	0.69	0.79	0.42	0.42

BIS= Behavioral Inhibition System; BAS= Behavioral Activation System; PAS= Physical Anhedonia Scale; SAS= Social Anhedonia Scale; CPS= Chemosensory Pleasure Scale; PANSS= Positive and Negative Syndrome Scale; PANSS PS= PANSS Positive Symptoms; PANSS NS =PANSS Negative Symptoms; PANSS GS= PANSS General Symptomatology; PANSS Tot= PANSS Total; CDSS= Calgary Depression Scale for Schizophrenia

** *p* < 0.01

* *p* < 0.05

### Linear and logistic regression analyses

Findings of linear regression analysis indicate that BAS and BDI play key roles in predicting physical, social, and chemosensory anhedonia in HC (Table 4). Similarly, CDSS and BAS play a key role in predicting physical anhedonia in the SZ group, while no significant predictors were found for social or chemosensory anhedonia (Table 5).

**Table 4:** Linear Regression Analysis Predictors of PAS, SAS, and CPS in Healthy Controls

Variables	B	SE	Odds Ratio (95% CI)	One-tailed *p*-value	Adjusted R2
**PAS**					
BDI	0.44	0.14	0.38 [(0.16) – (0.7)]	0.003*	0.2
BAS	-0.36	0.21	-0.21 [(-0.78) – (0.05)]	0.09	
**SAS**					
BDI	0.39	0.12	0.38 [(0.14) – (0.63)]	0.002*	0.2
BAS	-0.26	0.19	-0.17 [(0.63) – (0.11)]	0.17	
**CPS**					
BDI	-0.37	0.14	-0.27 [(-0.65) – (-0.08)]	0.013*	0.37
BAS	0.99	0.22	0.49 [(0.56) –(1.43)]	< 0.001**	

BAS= Behavioral Activation System; PAS= Physical Anhedonia Scale; SAS= Social Anhedonia Scale; CPS= Chemosensory Pleasure Scale; BDI= Beck Depression Inventory

CI: confidence interval

** *p* < 0.01

* *p* < 0.05

**Table 5:** Linear Regression Analysis Predictors of PAS, SAS, and CPS in Schizophrenia

Variables	B	SE	Odds Ratio (95% CI)	One-tailed *p*-value	Adjusted R2
**PAS**					
CDSS	0.75	0.35	0.30 [(0.58) – (1.44)]	0.034*	0.07
BAS	-0.28	0.15	-0.25 [(-0.58) – (0.023)]	0.07	
**SAS**					
CDSS	0.28	0.34	0.12 [(-0.4) – (0.96)]	0.41	0.0048
BAS	0.13	0.15	0.13 [(-0.16) – (0.42)]	0.37	
**CPS**					
CDSS	0.18	0.31	0.03 [(-0.45) – (0.81)]	0.57	0.025
BAS	0.20	0.14	0.22 [(-0.07) – (0.47)]	0.15	

BAS= Behavioral Activation System; PAS= Physical Anhedonia Scale; SAS= Social Anhedonia Scale; CPS= Chemosensory Pleasure Scale; CDSS= Calgary Depression Scale for Schizophrenia

CI: confidence interval

* *p* < 0.05

Logistic regression analyses were conducted to examine whether BAS and different dimensions of anhedonia predicted schizophrenia group membership.

In the first model including BAS and PAS, the overall model was statistically significant (χ² = 14.24, *p* < 0.001), explaining 15% of the variance (Nagelkerke R² = 0.15). The model correctly classified 66.7% of the total sample and 60.7% of patients. The Hosmer–Lemeshow test indicated a good model fit (*p* = 0.34).

In the second model, which included BAS and SAS, the model was also significant (χ² = 13.80, *p* < 0.001), accounting for 14% of the variance (Nagelkerke R² = 0.14). The overall classification accuracy was 65%, and the model correctly identified 60.7% of patients. The Hosmer–Lemeshow test indicated an adequate fit (*p* = 0.83). In the third model, which included BAS and CPS, the overall model was significant (χ² = 11.70, *p* = 0.003), explaining 12% of the variance (Nagelkerke R² = 0.12). The model correctly classified 64.2% of the total sample and 55.7% of patients, with an acceptable fit according to the Hosmer–Lemeshow test (*p* = 0.051).

Details of regression coefficients, Wald statistics, and odds ratios are presented in Table 6.

**Table 6:** Logistic Regression Models Predicting Schizophrenia Group Membership

Predictor Variable						
	B	SE	Wald	*p*	Odds Ratio (%95 CI)	Nagelkerke R2
Model 1						
BAS	-0.08	0.032	6.74	0.09*	0.92 [(0.87) – (0.98)]	0.15
PAS	0.04	0.023	3.23	0.072	1.04 [(0.99) – (1.09)]	
Model 2						
BAS	0.09	0.03	8.76	0.003*	0.91 [(0.86) – (0.97)]	0.14
SAS	0.04	0.02	2.80	0.09	1.04 [(0.99) – (1.09)]	
Model 3						
BAS	-0.09	0.03	6.70	< 0.01**	0.92 [(0.86) – (0.98)]	0.12
CPS	-0.02	0.02	0.77	0.38	0.98 [(0.95) – (1.02)]	

BAS = Behavioral Activation System; PAS = Physical Anhedonia Scale; SAS = Social Anhedonia Scale; CPS = Chemosensory Pleasure Scale; CI = Confidence Interval

** *p* < 0.01

* *p* < 0.05

## Discussion

This study examined the combined role of BIS/BAS systems, together with depressive symptoms, in predicting anhedonia among patients with SZ compared to HC. Results revealed that SZ patients exhibited significantly lower BAS activity than HCs, while no significant difference in BIS activity was observed between groups. SZ patients also reported higher levels of anhedonia across multiple domains. In HCs, both BAS activity and depressive symptoms significantly predicted anhedonia, whereas this relationship was absent in SZ.

Consistent with prior research identifying motivational deficits as a hallmark of schizophrenia, our findings indicate lower BAS activity in SZ patients compared to HCs. BAS, a central construct in Gray’s Reinforcement Sensitivity Theory, regulates reward sensitivity and goal-directed behaviour [30]. Behavioural activation and depressive symptomatology have been both theoretically and empirically linked to anhedonia [31]. Reduced BAS activity may therefore reflect diminished drive to pursue rewarding outcomes, contributing to avolition and anhedonia in SZ [32].

Interestingly, while BIS and BAS were negatively correlated in healthy controls, a positive correlation emerged in the SZ group. This divergence may indicate dysregulated motivational systems in schizophrenia, in which approach and avoidance tendencies are co-activated rather than balanced, reflecting disrupted integration of reward- and threat-related processing [5, 43]. Such dysregulation may explain the coexistence of motivational deficits and anxiety symptoms in some patients. The absence of significant differences in BIS activity between groups further suggests that punishment sensitivity and inhibitory control mechanisms remain relatively intact in SZ [34]. This contrasts with previous reports of heightened BIS activity in schizophrenia, particularly among patients with anxiety-related symptoms [36, 37]. These inconsistencies likely stem from heterogeneity in clinical subtypes, illness severity, and methodological approaches to measuring BIS.

The literature on punishment sensitivity and avoidance learning in schizophrenia remains mixed. Some studies using reinforcement-learning paradigms report reduced sensitivity to punishment or negative feedback, suggesting blunted avoidance motivation [33], whereas others find increased punishment sensitivity [34] or preserved inhibition under anxiety [11]. The present findings support the notion that motivational dysfunction in SZ is primarily associated with diminished reward responsiveness rather than heightened punishment sensitivity. Future studies integrating behavioural and neuroimaging measures are needed to clarify whether altered punishment sensitivity reflects a trait-level imbalance or task-specific learning deficit.

Motivational impairments contribute substantially to functional disability in SZ, including reduced engagement in rewarding activities and diminished goal-directed behaviour [35]. Our results differ from previous studies reporting increased BIS activity in patients with schizophrenia, particularly those with anxiety-related symptoms [36, 37]. These differences may be due to heterogeneity in the schizophrenia spectrum. At the same time, methodological differences such as symptom severity, clinical subtypes, or measurement tools used to evaluate BIS may also have played a role in contradictory results. Therefore, these findings underscore that motivational deficits in schizophrenia may be specific to reward-related processing rather than generalized deficits in behavioural regulation.

In the current study, patients with schizophrenia rated significantly higher on both the PAS and the SAS, reflecting higher levels of self-reported impairments in pleasure associated with physical and social experiences compared to healthy controls. These results are in line with previous studies showing higher levels of physical and social anhedonia in schizophrenia [38]. On the other hand, no significant group difference was found on the CPS, suggesting relatively intact pleasure associated with chemosensory experiences such as taste and smell [27]. However, since PAS, SAS, and CPS do not specifically differentiate between anticipatory and consummatory pleasure components, any interpretations regarding the specific temporal or motivational aspects of pleasure should be done with caution and restricted to the experiential elements of pleasure as measured by these scales.

BAS activity and depressive symptoms were significant predictors of anhedonia in healthy controls but not in SZ. Different depression measures were applied for each group (CDSS for SZ; BDI for HC) to ensure clinical validity. The minimal depressive symptoms reported by SZ patients may explain the absence of significant associations with anhedonia. Future research using identical depressive symptom measures across groups could enhance comparability.

The relationship between BAS activity, depressive symptoms, and anhedonia in HCs aligns with theoretical models positing that reduced behavioural activation lowers reward sensitivity, while depressive symptoms further diminish motivation and pleasure [9, 10]. This interaction likely produces a self-reinforcing cycle in which anhedonia perpetuates motivational deficits. However, in SZ, anhedonia may arise from distinct neurobiological mechanisms. Unlike mood-related anhedonia observed in nonclinical populations, anhedonia in schizophrenia is considered a core negative symptom associated primarily with deficits in anticipatory rather than consummatory pleasure [4, 37]. These deficits are thought to result from impairments in dopaminergic reward processing independent of mood-related pathology.

Although anhedonia is a primary symptom of schizophrenia, previous results on various aspects of pleasure have been inconsistent [4, 8, 38–41]. This is likely due to differences in symptom severity, measurement techniques, and population characteristics. Instead of focusing on the direct distinction between anticipatory and consummatory pleasure, the current results emphasize domain-specific effects of anhedonia in physical, social, and chemosensory contexts. Thus, the current results should be viewed as indicating domain-specific differences in pleasure alteration rather than conclusive evidence for selective impairment in various aspects of pleasure.

The finding that BAS activity and depressive symptoms jointly predicted anhedonia in HCs—but not in SZ—highlights distinct motivational mechanisms in schizophrenia. In HCs, higher BAS activity correlated with lower anhedonia, consistent with preserved reward sensitivity [42]. In SZ, by contrast, dysregulated approach–avoidance balance may disrupt this relationship, leading to motivational impairment even in the absence of depressive symptomatology. This divergence may indicate dysregulated motivational systems in schizophrenia, where approach and avoidance tendencies are co-activated rather than balanced, reflecting disrupted integration of threat- and reward-related processing [5, 43].

The present findings have several clinical implications. For individuals with depressive symptoms and anhedonia, interventions such as cognitive-behavioural therapy and behavioural activation targeting BAS dysregulation may enhance reward sensitivity and motivation [44]. In schizophrenia, treatment strategies should focus on the underlying neurobiological and behavioural mechanisms of motivational dysfunction. Pharmacological interventions addressing dopaminergic abnormalities, combined with psychosocial treatments such as cognitive remediation and goal-directed behavioural activation, may improve motivation and reward processing [8, 45]. Moreover, understanding the relative preservation of BIS functioning in SZ may aid in managing anxiety symptoms without exacerbating motivational deficits. A comprehensive therapeutic approach targeting both reward and avoidance systems could significantly improve quality of life in SZ.

Several limitations should be acknowledged. First, the cross-sectional design precludes causal inferences regarding relationships among BIS/BAS activity, depressive symptoms, and anhedonia. Longitudinal studies are needed to determine whether motivational deficits are stable traits or progressive phenomena. Second, this study represents the first application of the BIS/BAS scale to a Turkish schizophrenia sample. As a self-report measure, BIS/BAS may be subject to bias; future research should incorporate experimental paradigms to corroborate these findings.

Although no significant differences in age or gender were found, group differences in education level and marital status may have influenced outcomes. The SZ sample exhibited relatively mild negative symptoms (mean PANSS Negative = 14; range 7–25), which may limit generalisability to more severely affected populations. Including participants with broader illness severity would enhance representativeness.

In healthy controls, depressive symptoms were within the non-clinical range, suggesting that observed variability in anhedonia primarily reflects individual differences in reward responsiveness rather than clinical depression. These results imply that subclinical reductions in pleasure may serve as dimensional markers of motivational functioning across populations. Future studies including clinically depressed cohorts are warranted to verify this interpretation.

Moreover, as participants were clinically stable outpatients, this restricted symptom range may limit the generalizability of the findings to patients with more severe negative or motivational deficits. Therefore, heterogeneity in schizophrenia—encompassing symptom profiles, illness duration, medication history, and cognitive functioning—may also have influenced results. Future research should consider these variables and utilise neuroimaging combined with behavioural assessments to elucidate the neurobiological mechanisms underlying BIS/BAS functioning and anhedonia in SZ.

## Conclusion

This study highlights the multifaceted nature of anhedonia in schizophrenia and its distinct underlying mechanisms compared with those of healthy controls. While BAS activity and depressive symptoms significantly predicted anhedonia in healthy individuals, anhedonia in SZ appeared to result from fundamental deficits in reward processing systems independent of mood-related mechanisms. These findings emphasize the importance of developing targeted therapeutic strategies that address specific motivational and neurobiological impairments in SZ.

Future research employing experimental techniques and pleasure measurement instruments tailored to distinguish between anticipatory and consummatory processes is required to elucidate the mechanisms underlying hedonic impairment in schizophrenia. Moreover, to better understand how the BAS and BIS interact, it is essential to investigate their roles in the development of anhedonia and anxiety within schizophrenia. Such investigations may pave the way for more effective, evidence-based interventions. Cognitive-behavioural techniques that promote engagement with rewarding activities, pharmacological interventions that enhance dopaminergic signalling within reward pathways, and psychoeducational programs addressing avoidance behaviours may all help to improve motivational functioning.

Integrating BIS/BAS assessment into clinical evaluation could enable more precise prediction of individual treatment response and allow clinicians to tailor interventions to the specific motivational profiles of patients with schizophrenia.

## Data Availability

Data are available on a reasonable request.
